# Prospective Multimodal Assessment of Radiation-Induced Subclinical Cardiac Changes in Patients with Left Breast Cancer Using Hematologic Biomarkers, Echocardiography, and ^18^F-FDG PET/CT: A Pilot Study

**DOI:** 10.3390/cancers18050811

**Published:** 2026-03-03

**Authors:** Yong Kyun Won, Jeong Won Lee, Sang Mi Lee, Ik Dong Yoo, Sun-pyo Hong, Eun Seog Kim, Bohyun Kim, Hee-Dong Kim, Jung Eun Kim, Sera Oh, Nam Hun Heo, Gyeonghee Yoo, In Young Jo

**Affiliations:** 1 Department of Radiation Oncology, Soonchunhyang University Cheonan Hospital, 31, Sooncheonhyang 6-gil, Dongnam-gu, Cheonan 31151, Republic of Korea; yong.won@schmc.ac.kr (Y.K.W.);; 2 Department of Nuclear Medicine, Soonchunhyang University Cheonan Hospital, 31, Sooncheonhyang 6-gil, Dongnam-gu, Cheonan 31151, Republic of Korea; 3 Department of Laboratory Medicine, Soonchunhyang University Cheonan Hospital, 31, Sooncheonhyang 6-gil, Dongnam-gu, Cheonan 31151, Republic of Korea; 4 Division of Cardiology, Department of Internal Medicine, Soonchunhyang University Cheonan Hospital, 31, Sooncheonhyang 6-gil, Dongnam-gu, Cheonan 31151, Republic of Korea; 105225@schmc.ac.kr; 5 Department of Dermatology, Soonchunhyang University Cheonan Hospital, 31, Sooncheonhyang 6-gil, Dongnam-gu, Cheonan 31151, Republic of Korea; 6 Industry-Academic Cooperation Foundation, Soonchunhyang University, 22, Soonchunhyang-ro, Asan 31538, Republic of Korea; 7 Clinical Trial Center, Soonchunhyang University Cheonan Hospital, 31, Sooncheonhyang 6-gil, Dongnam-gu, Cheonan 31151, Republic of Korea; hello3933@schmc.ac.kr; 8 Division of Cardiology, Department of Pediatrics, Soonchunhyang University Cheonan Hospital, 31, Sooncheonhyang 6-gil, Dongnam-gu, Cheonan 31151, Republic of Korea

**Keywords:** radiation therapy, breast cancer, cardiotoxicity, laboratory test, echocardiography, PET/CT

## Abstract

Background/Objectives: This prospective study aimed to investigate asymptomatic microscopic changes in the myocardium following postoperative radiation therapy (RT) in patients with left breast cancer using multi-medical assessment techniques. Methods: This study included 16 left-sided breast cancer patients who received postoperative RT between January 2021 and December 2022 at our institution. Cardiac examinations were performed before RT and at 1, 12, 24, and 48 weeks after RT. We conducted comparative analyses between pre-RT and various post-RT time points, exploring correlations between changes in hematologic biomarkers, global longitudinal strain (GLS), and myocardial metabolism. Results: Inflammatory biomarkers such as the neutrophil–lymphocyte, platelet–lymphocyte, and lymphocyte–monocyte ratios changed between the pre- and post-RT periods but returned to normal levels after several months. However, troponin T and soluble suppression of tumorigenicity 2 showed sustained changes during the 1-year follow-up period. Among echocardiographic parameters, GLS_LAX showed a significant difference between pre-RT and post-RT assessments. Additionally, irradiated and non-irradiated myocardial metabolic ratios on ^18^F-fluorodeoxyglucose positron emission tomography/computed tomography differed between pre-RT and post-RT and remained altered up to one year after treatment. Conclusions: These findings suggest that subclinical myocardial changes may persist following RT, although the clinical significance of subclinical myocardial changes remains uncertain and warrants further investigation.

## Simple Summary

Radiation therapy after breast-conserving surgery is an essential treatment for breast cancer, but in left-sided disease the heart may be exposed to radiation, raising concerns about cardiac toxicity. Although clinically apparent heart disease usually develops years after treatment, subtle myocardial changes may occur much earlier without symptoms. In this prospective pilot study, we evaluated early subclinical cardiac changes in patients with left-sided breast cancer treated with postoperative radiation therapy without chemotherapy. Using blood biomarkers, echocardiography, and ^18^F-FDG PET/CT, we assessed inflammatory, functional, and metabolic myocardial changes over one year. We observed transient alterations in cardiac biomarkers, persistent metabolic changes in irradiated myocardial regions, and subtle strain abnormalities despite preserved cardiac function and absence of clinical symptoms. These findings suggest that microscopic radiation-induced myocardial changes can persist for at least one year and highlight the importance of early cardiac monitoring and strategies to reduce cardiac radiation exposure in breast cancer radiation therapy.

## 1. Introduction

Postoperative radiation therapy (RT) following breast-conserving surgery (BCS) is a standard treatment for breast cancer patients [1]. Although RT has recently been omitted in highly selected patient groups [2], BCS followed by RT has generally been associated with favorable oncologic outcomes comparable to total mastectomy, while allowing breast preservation and is associated with favorable cosmetic outcomes, preserved physical function, and improved quality of life compared with total mastectomy.

However, postoperative RT for breast cancer can cause several side effects, including cardiac toxicity [3,4]. The localized nature of RT leads to variability in the types and severity of side effects between the right and left breasts. Notably, for patients with left-sided breast cancer, RT may have a more significant impact on the myocardium than it does for those with cancer in the contralateral breast [5].

Common radiation-induced cardiac toxicities include myocarditis, coronary artery disease, arrhythmias, cardiomyopathy, and valvular disease, among others [6,7]. These cardiac symptoms typically appear 3–10 years post-RT, with some emerging decades later [5,8]. Nevertheless, prior research conducted by our group has indicated that microscopic changes in the myocardium without symptoms can begin as early as 2–3 months following the completion of RT [9]. Our previous retrospective study compared left- and right-sided breast cancer patients and demonstrated a dose-dependent increase in myocardial FDG uptake in left-sided patients [9]. However, the analysis was limited to metabolic imaging alone. To address this limitation, the present prospective pilot study was intentionally limited to left-sided breast cancer patients who did not receive chemotherapy to more clearly isolate subclinical myocardial effects attributable specifically to RT. To this end, we performed laboratory tests, echocardiography, and ^18^F-fluorodeoxyglucose positron emission tomography/computed tomography (^18^F-FDG PET/CT) scans to confirm hematologic, functional, and metabolic changes in the myocardium following breast RT.

## 2. Methods

### 2.1. Patient Selection

This study enrolled patients with left-sided breast cancer who underwent postoperative RT at our institution from January 2021 to December 2022. The study was approved by the Institutional Review Board of Soonchunhyang University Cheonan Hospital (Approval No: SCHCA 2021-03-038) and was conducted in accordance with the revised Helsinki Declaration of 2013 and its subsequent amendments. Informed consent was obtained from each patient at least one week before the study began, and the consent process was managed according to the guidelines of the aforementioned Institutional Review Board. This study was also registered with the Clinical Research Information Service (CRiS), an international clinical trial registry platform (ICTRP) operated by the Korea Disease Control and Prevention Agency, under registration number KCT0006090 (first trial registration date: 22 April 2021).

The inclusion criteria were as follows: patients who (1) were aged 20–70 years, (2) were diagnosed with left-sided breast cancer, (3) had an Eastern Cooperative Oncology Group performance status of 0 or 1, (4) showed no evidence of metastasis at the time of diagnosis, (5) underwent BCS, (6) were scheduled for RT confined to the breast only, (7) had no medical history of cardiac disease, and (8) had not received prior chemotherapy. The exclusion criteria were as follows: patients who (1) had bilateral breast cancer, (2) underwent mastectomy, (3) had a history of RT to the thorax, (4) had other concomitant malignancies, or (5) were scheduled for axillary and/or supraclavicular RT.

A total of 20 patients were enrolled, and 16 were analyzed after 4 patients withdrew during the study.

### 2.2. Adjuvant RT

All patients underwent simulation CT with a 3-mm slice thickness using a Philips Brilliance Big Bore (Philips Medical Systems, Madison, WI, USA). They were positioned supine on a 15-degree tilting breast board (CIVCO Medical Solutions, Orange City, IA, USA), with both arms raised above their heads to ensure optimal breast exposure. CT imaging was performed with free-breathing and contrast-enhanced scans.

As in the previous study, the treatment plan utilized four portals with two opposing tangential angles using the field-in-field technique [9]. Physical wedges were applied along the x- and y-axes at each portal to enhance target dose conformity. Radiation treatment planning was conducted using Eclipse ver. 16.01.10 (Varian Medical Systems, Palo Alto, CA, USA) with a 6-MV photon beam. The entire breast received 50 Gy in 25 fractions over 5 weeks, with a tumor bed boost of 10 Gy in 5 fractions.

### 2.3. Pre- and Post-RT Myocardial Examination

Pre- and post-RT myocardial examinations included laboratory tests, echocardiography, and ^18^F-FDG PET/CT scans.

Laboratory tests were performed before RT (Pre-RT), one week after RT completion (Post-1), and at 12, 24, and 48 weeks (Post-2, Post-3, and Post-4), for a total of five assessments. Hematologic inflammatory biomarkers such as neutrophil–lymphocyte ratio (NLR), platelet–lymphocyte ratio (PLR), and monocyte-lymphocyte ratio (MLR) were evaluated. Cardiac-specific biomarkers, including high-sensitivity troponin T (hs-TnT, ≤0.014 ng/mL) and N-terminal prohormone of brain natriuretic peptide (NT-pro BNP, ≤130 pg/mL), were also assessed. High-sensitivity troponin T (hs-TnT) levels were measured using a Cobas^®^ e801 electrochemiluminescence immunoassay (Roche Diagnostics, Mannheim, Germany) and the soluble suppression of tumorigenicity 2 (sST2, ng/mL) was analyzed using an enzyme-linked immunosorbent assay (ELISA) with DST200 (Human ST2/IL-33R Quantikine ELISA Kit, R&D Systems, Minneapolis, MN, USA).

^18^F-FDG PET/CT scans were performed using the same protocol as described in our previous study [9]. Metabolic activity of the left ventricular myocardium was evaluated using MIM software version 6.8.10 (MIM Software, Beachwood, OH, USA). For initial registration, the CT images from PET/CT were translated and rotated to align with the simulation CT using the sternum and ipsilateral ribs as anatomical landmarks. Subsequently, a second fine-registration step was performed, in which a radiation oncologist manually reviewed and refined the alignment based on cardiac position, morphology, and orientation. ^18^F-FDG PET/CT scans were acquired for research purposes at three time points: before RT (Pre-RT), 12 weeks after RT completion (Post-2), and 48 weeks after RT completion (Post-4). On the basis of previous reports, two thresholds were applied: 30 Gy, a dose level frequently associated with cardiac toxicity, and 47.5 Gy, representing the high-dose region near the prescription dose shown to affect myocardial metabolism and function [10,11]. Accordingly, four groups were defined: myocardium irradiated with more than 30 Gy (irradiated myocardium of 30), less than 30 Gy (non-irradiated myocardium of 30), more than 47.5 Gy (irradiated myocardium of 47.5), and less than 47.5 Gy (non-irradiated myocardium of 47.5). The FDG uptake of the entire and irradiated myocardium was measured using OsiriX MD (Pixmeo, Geneva, Switzerland). The mean SUV of the blood pool was measured using a spheroid-shaped volume-of-interest, which was drawn over the descending aorta. The myocardium-to-blood pool uptake ratio was calculated from the mean SUVs of the myocardium and the blood pool. Additionally, the uptake ratio between the irradiated and non-irradiated myocardium was calculated using the mean and maximum SUV values.

Echocardiography was performed before RT (Pre-RT) and at 12 weeks (Post-2), 24 weeks (Post-3), and 48 weeks (Post-4) after RT completion, for a total of four times, to assess the ejection fraction (EF, %) and average global longitudinal strain (GLS, %). We also analyzed the subgroups of GLS: long axis GLS (GLS_LAX, %), apical 4 chamber GLS (GLS_A4C, %), and apical 2 chamber GLS (GLS_A2C, %).

### 2.4. Statistical Analyses

The statistical analysis was conducted in two steps. Initially, we examined changes in hematologic, inflammatory, and cardiac-specific biomarkers, EF, GLSs, and metabolic activity from pre-RT to Post-4. We subsequently assessed the differences between pre-RT and the subsequent time points (Post-1 through Post-4) to analyze changes at each interval. For continuous variables, we employed Student’s *t*-test, paired *t*-test, and one-way repeated measures analysis of variance with post hoc pairwise multiple comparisons and applied the Bonferroni correction for multiple comparisons. All the statistical tests were two-tailed and conducted using SPSS software (Version 27.0, IBM Corp., Armonk, NY, USA), and *p*-values < 0.05 were considered statistically significant. Given the exploratory and pilot nature of this study, no a priori power calculation was performed.

## 3. Results

### 3.1. Patient Characteristics

The study enrolled 20 patients, 4 of whom withdrew, leaving 16 patients for analysis. All participants were female, with a median age of 47 years (range: 25–65). Among these patients, two had hypertension, one had type II diabetes mellitus, and none had hyperlipidemia. The mean body mass index was 23.1 (range: 18.4–32.5). Thirteen patients were diagnosed with ductal carcinoma in situ, while three had invasive ductal carcinoma. All patients underwent an RT regimen of 50 Gy in 25 fractions to the entire breast and an additional 10 Gy in 5 fractions directed at the tumor bed (Table 1). All patients experienced no cardiac events or clinical symptoms during the 1-year follow-up period.

### 3.2. Laboratory Tests

**Figure 1:**
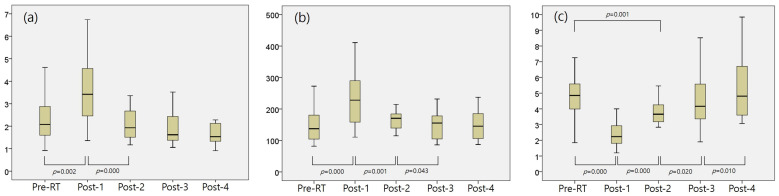
Changes in hematologic inflammatory biomarkers before and after breast RT; (**a**) neutrophil-lymphocyte ratio, (**b**) platelet-lymphocyte ratio, (**c**) lymphocyte-monocyte ratio. There were significant changes in all the inflammatory biomarkers before and after breast RT, and they reverted to baseline levels from 3 months to 1 year.

The results of the correlation analyses for the NLR, PLR, and MLR are presented in Figure 1 and Supplementary Table S1. The NLR significantly increased from 2.28 ± 0.96 to 3.54 ± 1.42 from Pre-RT to Post-1 (*p* = 0.002) and then decreased from 3.54 ± 1.42 to 2.07 ± 0.72 from Post-1 to Post-2 (*p* = 0.000). No significant differences were detected between Pre-RT and Post-2, Post-3, or Post-4. PLR significantly changed from Pre-RT (143.88 ± 50.06) to Post-1 (236.84 ± 88.40) (*p* = 0.000), from Post-1 to Post-2 (169.15 ± 39.96) (*p* = 0.001), and from Post-2 to Post-3 (150.83 ± 44.69) (*p* = 0.043). While a minor difference was noted between Pre-RT and Post-2 (*p* = 0.055), no significant differences were found between Pre-RT and Post-2 or Post-4. The MLR significantly changed at all intervals: Pre-RT versus Post-1 (4.99 ± 1.68 vs. 2.56 ± 1.20, *p* = 0.000), Post-1 versus Post-2 (2.56 ± 1.20 vs. 3.76 ± 1.04, *p* = 0.000), Post-2 versus Post-3 (3.76 ± 1.04 vs. 4.57 ± 1.77, *p* = 0.020), and Post-3 versus Post-4 (4.57 ± 1.77 vs. 5.24 ± 2.04, *p* = 0.010). A significant difference was observed between Pre-RT and Post-2 (*p* = 0.001), but not between Pre-RT and Post-3 or Post-4.

**Figure 2:**
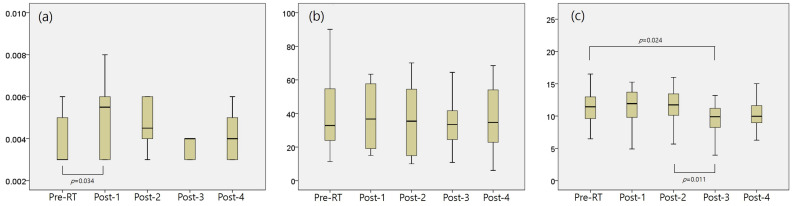
Changes in cardiac-specific biomarkers before and after breast RT; (**a**) troponin T (TnT), (**b**) N-terminal prohormone of brain natriuretic peptide (NT-pro BNP), (**c**) soluble suppression of tumorigenicity 2 (sST2). There were significant changes in all cardiac-specific biomarkers before and after breast RT, except for NT-pro BNP. TnT increased immediately and subsequently recovered, whereas sST2 showed the most substantial changes at 6 months post-RT and slightly recovered at the 1-year follow-up.

Cardiac-specific biomarkers, including TnT, NT-pro BNP, and sST2, are discussed in Figure 2 and Supplementary Table S1. TnT exhibited significant differences solely between Pre-RT and Post-1 (0.0043 ± 0.0026 vs. 0.0052 ± 0.0024, *p* = 0.034), with no notable variations at subsequent time points. NT-pro BNP showed no significant changes at any time point. Conversely, sST2, indicative of prominent cardiac damage, demonstrated significant differences between Post-2 and Post-3 (11.91 ± 3.92 vs. 10.57 ± 4.51, *p* = 0.011), and between Pre-RT and Post-3 (*p* = 0.024), with a marginal difference between Pre-RT and Post-4 (*p* = 0.052). Like other hematologic inflammatory biomarkers, such as the NLR, PLR, and MLR, TnT and sST2 levels fluctuated after breast RT, with TnT increasing immediately and subsequently recovering, whereas sST2 showed the most substantial changes at 6 months post-RT and slight recovery at the 1-year follow-up.

### 3.3. ^18^F-FDG PET/CT

**Figure 3:**
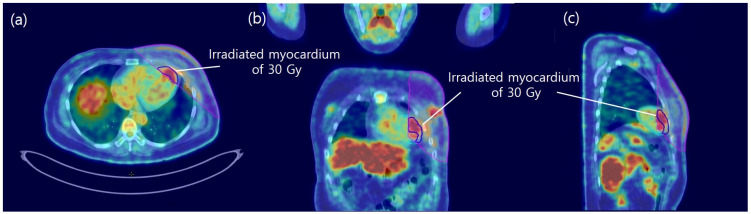
Images of ^18^F-FDG PET/CT 12 weeks after RT completion in a 46-year-old woman with left breast cancer (**a**–**c**). An irradiated area more than 30 Gy is indicated by the pink thin solid line; A segment with an irradiated myocardium more than 30 Gy (irradiated myocardium of 30) is indicated by the blue thick solid line. Less than 30 Gy (non-irradiated myocardium of 30) is indicated by the cyan thin solid line. The metabolic activity of the ‘irradiated myocardium of 30’ is substantially different from that of the ‘non-irradiated myocardium of 30’ after RT.

**Figure 4:**
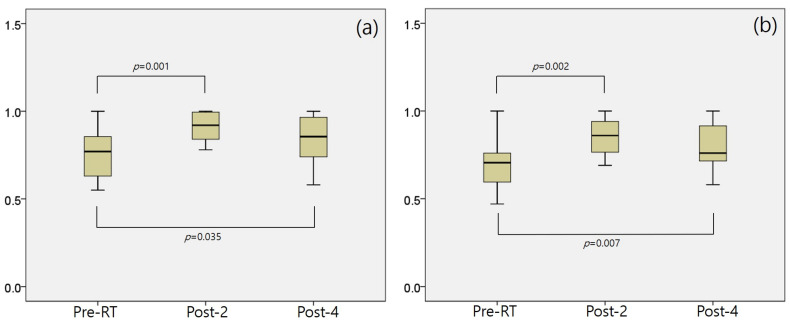
Changes in the SUVmax on ^18^F-FDG PET/CT before and after breast RT; the ratio of irradiated myocardium to non-irradiated area receiving more than 30 Gy (**a**) and 47.5 Gy (**b**). In both groups, statistically significant increases were observed in Pre-RT and Post-2, and between Pre-RT and Post-4, whereas no significant differences were found between Post-2 and Post-4. These findings suggest that metabolic changes in regions receiving more than 30 Gy may persist for up to one year after RT.

The myocardium-to-blood pool uptake ratios are summarized in Table 2. Although the mean ratio decreased over time, none of these changes were statistically significant. The ratio of irradiated myocardium to non-irradiated area receiving more than 30 Gy or 47.5 Gy was calculated for Pre-RT, Post-2, and Post-4. (Figure 3 and Supplementary Table S2) Significant changes in the SUVmax were observed in both the 30 Gy and 47.5 Gy groups from Pre-RT to Post-2 (0.76 ± 0.14 vs. 0.91 ± 0.14, *p* = 0.001 for 30 Gy and 0.71 ± 0.15 vs. 0.85 ± 0.13, *p* = 0.002 for 47.5 Gy) and from Pre-RT to Post-4 (0.76 ± 0.14 vs. 0.85 ± 0.13, *p* = 0.035 for 30 Gy and 0.71 ± 0.15 vs. 0.79 ± 0.14, *p* = 0.007 for 47.5 Gy), with no significant differences between Post-2 and Post-4 at either 30 Gy or 47.5 Gy (Figure 4). These findings suggest that metabolic alterations in myocardial regions receiving more than 30 Gy may persist for up to one year after RT.

### 3.4. Echocardiography

**Figure 5:**
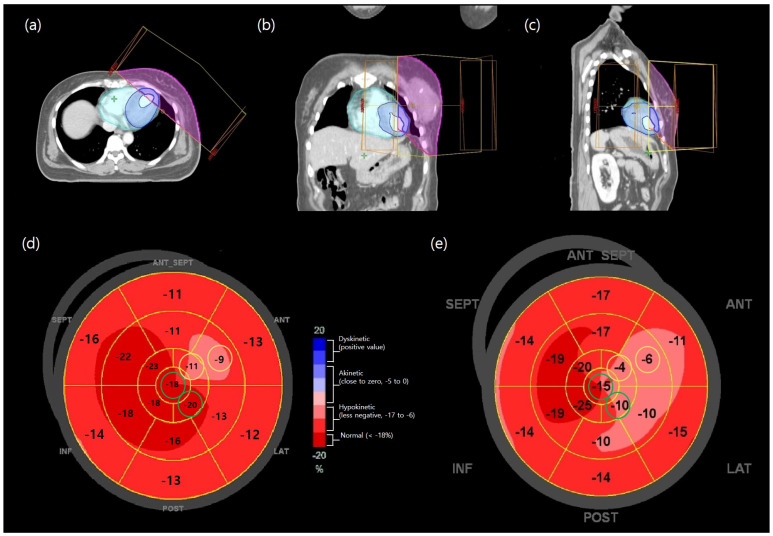
Axial, coronal, and sagittal images of RT planning (**a**–**c**) of a 46−year−old woman with left breast cancer and her bull’s eye plot of echocardiography before (**d**) and 12 weeks after (**e**) RT completion; the heart is indicated by the cyan translucent area; the blue translucent area indicates the whole left ventricular myocardium; the irradiated area more than 30 Gy is indicated by the pink translucent area; and the irradiated myocardium more than 30 Gy (irradiated myocardium of 30 Gy) is indicated by the blue thick solid line (**a**–**c**). The segments representing the normal strain before RT (**d**, green circles) were changed to hypokinetic (**e**, green circles) 3 months after RT, and the segments representing the hypokinetic strain (**d**, yellow circles) were changed to akinetic (**e**, yellow circles). The changed segments are mainly the apex, apical−anterior, and mid-anterior regions, corresponding to the irradiated myocardium of the 30 Gy.

We investigated whether echocardiography could detect functional changes even in the presence of only microscopic myocardial damage after breast RT. There were no differences in EF or GLS across all follow-up periods. However, subgroup analysis of GLS revealed a significant decrease in the absolute value of GLS_LAX from Pre-RT to Post-2 (−20.20 ± 2.65 vs. −18.89 ± 2.61, *p* = 0.015). (Figure 5 and Supplementary Table S3) No significant changes were observed in the other subgroups (GLS_A4C and GLS_A2C) throughout the follow-up period.

## 4. Discussion

Radiation-induced myocardial toxicity remains a critical and significant side effect in left-sided breast cancer patients. Radiation exposure can impair myocardial microvasculature, leading to ischemic and fibrotic alterations; however, clinically overt cardiac disease often develops years after treatment [12]. This latency highlights the importance of identifying early, asymptomatic myocardial changes following RT. Despite several studies evaluating radiation-related myocardial changes, comprehensive investigations integrating hematologic, functional, and metabolic assessments remain limited [13,14]. Accordingly, this study aimed to explore subclinical myocardial changes after RT using a multimodal approach.

Radiation exposure induces oxidative stress and inflammatory responses in myocardial tissue [15,16]. Hematologic inflammatory biomarkers, including NLR, PLR, and LMR, have been used to assess cardiovascular risk and prognosis [17,18,19]. In these studies, the NLR, PLR, and LMR were monitored before and after RT, with the most significant changes in these biomarkers noted immediately after RT completion and substantial recovery observed over the 3- to 6-month period. This was similarly confirmed in our study. In the present study, these biomarkers showed transient changes shortly after RT, with the most pronounced alterations observed at 1 week after treatment completion, followed by gradual recovery over several months. Although the timing of recovery differed among NLR, PLR, and LMR, all biomarkers returned to pre-RT levels within one year, suggesting that systemic inflammatory responses to RT appear to be largely reversible within the observed follow-up period. These findings highlight the limited utility of hematologic inflammatory biomarkers as long-term indicators of localized subclinical myocardial changes following RT. A recent study has suggested that cardiotoxicity may occur even after RT for tumors located distant from the heart [20]. However, these observations were not derived from RT alone but rather reflected the cumulative effects of multiple cancer treatment modalities and, therefore, do not specifically address the direct impact of RT. Nevertheless, as proposed in these studies, it cannot be completely excluded that RT, even in the absence of direct cardiac irradiation, may increase cardiovascular risk through the induction of systemic inflammatory responses. Further studies with more refined and modality-specific designs are warranted to delineate the independent contribution of RT to such systemic cardiovascular effects.

TnT levels peaked one week after RT completion and subsequently declined by 3–6 months, suggesting transient myocardial injury during the period of radiation exposure. TnT rapidly leaks from myocardial cells into the bloodstream shortly after myocardial injury and can persist in the blood for 10 to 21 days [21]. In this study, TnT levels increased after RT but decreased by the 3- to 6-month assessment, suggesting that myocardial injury may have occurred during the period of radiation exposure. If TnT levels remain elevated beyond 3 months after RT, this could indicate ongoing myocardial damage and warrants further investigation. While normalization of TnT levels may reflect the absence of additional myocardial damage, it does not signify recovery of the damaged myocardium.

NT-proBNP primarily reflects myocardial wall stress and volume overload rather than direct myocardial injury and is therefore not a specific marker of radiation-induced myocardial damage [22]. Although the synthesis and secretion of NT-pro BNP occur in myocardial cells, its degradation occurs within 90–120 min, making it difficult to detect changes unless sustained stress is present [22]. In our study, NT-pro BNP levels did not significantly change during follow-up, and all values remained within the normal range, suggesting the absence of clinically relevant hemodynamic stress within 12 months after RT. Although previous studies have reported NT-proBNP elevation after left-sided breast RT in patients receiving higher cardiac doses [23], our findings indicate that its role may be limited in detecting radiation-related subclinical myocardial changes, serving mainly as supportive information on functional stress.

The IL-33/sST2 signaling pathway has been implicated in myocardial remodeling and cardioprotection. In non-damaged hearts, fibroblasts produce interleukin (IL)-33, a type of cytokine, following pro-inflammatory stimulation [24]. It binds to the transmembrane form of ST2 (ST2L) on cardiomyocyte membranes [25,26,27]. Following this binding, several intracellular cascades are triggered, culminating in the upregulation of NF-kB, which inhibits fibrosis and hypertrophy of the myocardium. However, sST2 competitively interferes with the binding of ST2L to IL-33 because of its structural similarity. Consequently, elevated sST2 levels impede the prevention of cardiac damage. Accordingly, lower sST2 levels may reflect a more favorable myocardial milieu for recovery. In the present study, sST2 levels showed minimal change during the first three months after RT and subsequently decreased, coinciding with the earlier transient elevation in TnT. This temporal pattern may indicate persistent myocardial stress during the early post-RT period, followed by a possible compensatory response over time. However, sST2 level remained lower than baseline up to one year after RT. These findings are temporally consistent with the duration of metabolic changes observed on ^18^F-FDG PET/CT in both our previous retrospective study and the present study, but further large-scale study is needed as to whether there is a correlation between the two parameters.

As in our previous study [9], the metabolic activity of the irradiated myocardium increased at 3 months after RT completion and remained elevated for up to one year. An increase in myocardial metabolic activity was associated with the irradiated dose, with higher uptake observed in regions receiving higher radiation doses, suggesting a dose-related pattern of myocardial metabolic alteration. In the present study, however, metabolic changes were more pronounced in the 30 Gy group. This may be explained by the larger myocardial volume encompassed within the 30 Gy region, allowing greater sensitivity for detecting metabolic changes, whereas regions receiving 47.5 Gy represent smaller volumes adjacent to the prescription dose [28,29]. In addition, higher-dose regions may undergo earlier fibrotic or necrotic changes with limited residual metabolic activity, whereas moderate-dose regions may exhibit more persistent inflammatory and metabolic responses [28,30]. While our previous retrospective study with a large cohort demonstrated clearer dose-dependent patterns, the limited sample size of this pilot study may have constrained our ability to reproduce these findings. Beyond conventional SUV-based measures, radiomic analysis of FDG-PET/CT may provide complementary information by capturing subtle metabolic heterogeneity not reflected by mean values alone. Nevertheless, the persistence of metabolic alterations in myocardial regions receiving more than 30 Gy for up to one year underscores the importance of minimizing cardiac radiation exposure. Although advanced RT techniques, such as intensity modulated RT or volumetric modulated arc therapy, may reduce myocardial radiation dose in patients with left-sided breast cancer. However, the relationship between asymptomatic myocardial changes and clinically overt myocardial injury has not been clearly established, and such techniques may increase low-dose radiation showers in breast cancer patients; therefore, careful consideration is required.

Myocardial microscopic changes were also assessed using echocardiography. GLS is a highly sensitive echocardiographic measurement that quantifies LV deformation along the long-axis from base to apex. It is commonly used to detect early subclinical LV dysfunction and is more sensitive than LVEF for identifying subtle systolic impairment [31,32,33]. In the present study, LVEF remained unchanged, whereas transient functional alterations were observed in GLS_LAX, among the GLS subgroups. Previous studies have reported that GLS changes are not reliably predicted by the mean heart dose [34]. Because myocardial strain includes longitudinal, circumferential, and radial components, impairment of longitudinal strain may occur without clinical symptoms, as circumferential strain can compensate for global systolic function [35,36]. GLS_LAX changes were observed when comparing irradiated and non-irradiated myocardial regions and were most apparent at 3 months after RT completion. Unlike A4C and A2C, which assess different wall segments and are more susceptible to inter-observer variability, GLS_LAX reflects longitudinal deformation along a single axis from apex to base and may therefore be more sensitive to subtle changes in anterior and apical regions receiving higher radiation doses. As shown in Figure 5, these irradiated regions included the apical to basal anterior and anterolateral segments, where corresponding strain alterations were identified. Although such changes were not observed in all patients, these findings suggest that evaluating region-specific myocardial strain may be more informative than relying solely on mean cardiac dose. However, given the limited sample size and the isolated significance of GLS_LAX, these results should be considered exploratory and interpreted with caution.

Additionally, in patients with cancer, myocardial changes may arise from the disease itself; cardiac involvement in these patients has been reported to predominantly affect circumferential rather than longitudinal strain [37]. In the present study, however, irradiated myocardium showed impairment in longitudinal strain, suggesting that strain alterations during cancer diagnosis and treatment may occur across multiple directions. Such cardiotoxic effects can influence long-term survival even after successful cancer treatment. Patients receiving thoracic RT may therefore be at increased risk of long-term cardiac effects, particularly in left-sided breast cancer, where the myocardium is directly exposed to radiation. Although effective interventions for asymptomatic subclinical myocardial damage have not yet been established, identifying early myocardial alterations remains important, and a multimodal approach may improve the prediction of radiation-induced heart disease.

According to the 2022 International Cardio-Oncology Society–Heart Failure (ICOS-HF) guidelines, breast cancer patients treated with left-sided RT without anthracyclines or HER2-targeted therapy are generally categorized as being at moderate risk of cardiotoxicity [38]. The guidelines highlight three main strategies to mitigate radiation-induced cardiotoxicity: (1) omission of RT, (2) reduction of irradiated dose and volume, and (3) use of advanced radiation techniques to minimize cardiac exposure. While omission of RT is rarely feasible in clinical practice, careful patient selection for accelerated partial breast irradiation and the use of intensity-modulated RT instead of three-dimensional conformal RT may further reduce cardiac exposure. These strategies emphasize the importance of individualized treatment planning to minimize long-term cardiac risk.

This study has several limitations, including a small sample size and a relatively short follow-up period. Because of the limited sample size, subgroup analyses according to comorbidities such as hypertension or diabetes mellitus could not be performed. Nevertheless, as a prospective study with relatively well-controlled and less heterogeneous patient characteristics, our findings provide preliminary evidence that comprehensive assessment of asymptomatic myocardial changes may contribute to understanding early radiation-related cardiac effects. The results should be interpreted with caution, and larger multicenter studies are warranted to validate these findings.

Another limitation is the relatively young age of the cohort. While this reflects the inclusion criteria excluding patients with prior cardiac disease, the lack of comparison with older, otherwise healthy patients may limit the applicability of the results to broader populations.

An additional limitation is the absence of cardiac MRI, the reference standard for myocardial structural and functional assessment. Due to limited research funding, accessibility, and the exploratory nature of this pilot study, cardiac MRI was not included. Furthermore, ^18^F-FDG PET/CT was performed under routine institutional conditions with standard fasting only, without specific myocardial metabolic preparation, which may have limited the accuracy of metabolic assessment. To address these limitations, a multimodal approach incorporating hematologic biomarkers, echocardiography, and metabolic imaging was used to provide complementary information on subclinical myocardial alterations. In future studies, cardiac magnetic resonance imaging should be incorporated to enable more comprehensive structural and tissue characterization, along with radiomics-based analysis of cardiac imaging in larger cohorts. In addition, long-term longitudinal follow-up of patients enrolled in the present study will be necessary to determine whether the observed asymptomatic myocardial changes translate into the development of clinically overt myocardial disease.

## 5. Conclusions

This prospective pilot study suggests a potential association between radiotherapy and early subclinical myocardial changes; however, the clinical significance of these findings requires further investigation.

## Figures and Tables

**Table 1:** Patients characteristics.

Characteristics	Value
Age	
Median (range)	47 (25–65)
Sex	
Male	0 (0%)
Female	16 (100%)
Pathology	
DCIS	13 (81.2%)
IDC	3 (18.8%)
High Blood Pressure	
Yes	2 (12.5%)
No	14 (87.5%)
Diabetes Mellitus	
Yes	1 (6.2%)
No	15 (93.8%)
Hyperlipidemia	
Yes	0 (0%)
No	16 (100%)
Body Mass Index	
Mean (range)	23.1 (18.4–32.5)
Total RT dose	
Whole breast	5000 cGy
Boost to tumor bed	1000 cGy
Cardiac dose-volume parameters	
Mean Heart Dose	655.71 cGy (404.5–1126.1)
V5	17.10% (7.98–29.59%)
V20	8.29% (3.23–18.68%)

DCIS, Ductal carcinoma in situ; IDC, Invasive ductal carcinoma; RT, Radiation therapy.

**Table 2:** The myocardium-to-blood pool uptake ratios in all patients (*n* = 16).

Patients	Myocardium-to Blood Pool Uptake Ratio		
	Pre-RT	Post-2	Post-4
All patients	1.36 ± 1.47	1.03 ± 0.70	0.84 ± 0.35
	Pre-RT vs. Post-2	Pre-RT vs. Post-4	Post-2 vs. Post-4
*p*-values	0.163	0.193	0.338

All data were expressed as average ± standard deviation. RT, radiation therapy; Post-2, 12 weeks after RT completion; Post-4, 48 weeks after RT completion.

## Data Availability

No primary data is available due to ethical restrictions.

## Supplementary Materials

The following supporting information can be downloaded at: https://www.mdpi.com/article/10.3390/cancers18050811/s1. Table S1: Changes in hematologic inflammatory and cardiac-specific biomarkers before and after breast RT; Table S2: Changes in metabolic activity on 18F-FDG PET/CT before and after breast RT; Table S3: Changes in global longitudinal strain on echocardiography before and after breast RT.

## Abbreviations

^18^F-FDG	Fluorine-18 fluorodeoxyglucose
A4C	Apical four-chamber view
A2C	Apical two-chamber view
BCS	Breast-conserving surgery
BNP	Brain natriuretic peptide
DCIS	Ductal carcinoma in situ
EF	Ejection fraction
GLS	Global longitudinal strain
hs-TnT	High-sensitivity troponin T
LMR	Lymphocyte-to-monocyte ratio
LV	Left ventricle
MLR	Monocyte-to-lymphocyte ratio
NLR	Neutrophil-to-lymphocyte ratio
NT-proBNP	N-terminal pro-brain natriuretic peptide
PET/CT	positron emission tomography/computed tomography
PLR	Platelet-to-lymphocyte ratio
RT	Radiation therapy
sST2	Soluble suppression of tumorigenicity 2
SUV	Standardized uptake value
V30/V47.5	Myocardial volume receiving ≥30 Gy/≥47.5 Gy
