# Supplementary material for: Prospective Multimodal Assessment of Radiation-Induced Subclinical Cardiac Changes in Patients with Left Breast Cancer Using Hematologic Biomarkers, Echocardiography, and 18F-FDG PET/CT: A Pilot Study
Source: Cancers (Basel). 2026 Mar 3;18(5):811. doi: 10.3390/cancers18050811 (PMC12984685; doi:10.3390/cancers18050811)
Supplement: Supplementary file 1 [file cancers-18-00811-s001.zip › cancers-4134335-supplementary.pdf]

Table S1. Changes in hematologic inflammatory and cardiac-specific biomarkers before and after breast RT

		Mean ± SD	p-value*
NLR	Pre-RT vs. Post-1	2.28 ± 0.96 vs. 3.54 ± 1.42	0.002
	Post-1 vs. Post-2	3.54 ± 1.42 vs. 2.07 ± 0.72	0.000
	Post-2 vs. Post-3	2.07 ± 0.72 vs. 1.91 ± 0.72	0.248
	Post-3 vs. Post-4	1.91 ± 0.72 vs. 1.86 ± 0.82	0.725
	Pre-RT vs. Post-2	2.28 ± 0.96 vs. 2.07 ± 0.72	0.447
	Pre-RT vs. Post-3	2.28 ± 0.96 vs. 1.91 ± 0.72	0.167
	Pre-RT vs. Post-4	2.28 ± 0.96 vs. 1.86 ± 0.82	0.131
PLR	Pre-RT vs. Post-1	143.88 ± 50.06 vs. 236.84 ± 88.40	0.000
	Post-1 vs. Post-2	236.84 ± 88.40 vs. 169.15 ± 39.96	0.001
	Post-2 vs. Post-3	169.15 ± 39.96 vs. 150.83 ± 44.69	0.043
	Post-3 vs. Post-4	150.83 ± 44.69 vs. 146.91 ± 46.12	0.428
	Pre-RT vs. Post-2	143.88 ± 50.06 vs. 169.15 ± 39.96	0.055
	Pre-RT vs. Post-3	143.88 ± 50.06 vs. 150.83 ± 44.69	0.511
	Pre-RT vs. Post-4	143.88 ± 50.06 vs. 146.91 ± 46.12	0.998
LMR	Pre-RT vs. Post-1	4.99 ± 1.68 vs. 2.56 ± 1.20	0.000
	Post-1 vs. Post-2	2.56 ± 1.20 vs. 3.76 ± 1.04	0.000
	Post-2 vs. Post-3	3.76 ± 1.04 vs. 4.57 ± 1.77	0.020
	Post-3 vs. Post-4	4.57 ± 1.77 vs. 5.24 ± 2.04	0.010
	Pre-RT vs. Post-2	4.99 ± 1.68 vs. 3.76 ± 1.04	0.001
	Pre-RT vs. Post-3	4.99 ± 1.68 vs. 4.57 ± 1.77	0.218
	Pre-RT vs. Post-4	4.99 ± 1.68 vs. 5.24 ± 2.04	0.498

Troponin T	Pre-RT vs. Post-1	0.0043 ± 0.0026 vs. 0.0052 ± 0.0024	0.034
	Post-1 vs. Post-2	0.0052 ± 0.0024 vs. 0.0049 ± 0.0020	0.510
	Post-2 vs. Post-3	0.0049 ± 0.0020 vs. 0.0044 ± 0.0023	0.088
	Post-3 vs. Post-4	0.0044 ± 0.0023 vs. 0.0046 ± 0.0024	0.423
	Pre-RT vs. Post-2	0.0043 ± 0.0026 vs. 0.0049 ± 0.0020	0.106
	Pre-RT vs. Post-3	0.0043 ± 0.0026 vs. 0.0044 ± 0.0023	0.684
	Pre-RT vs. Post-4	0.0043 ± 0.0026 vs. 0.0046 ± 0.0024	0.237
NT-Pro BNP	Pre-RT vs. Post-1	42.01 ± 25.79 vs. 36.77 ± 18.34	0.352
	Post-1 vs. Post-2	36.77 ± 18.34 vs. 39.31 ± 29.19	0.704
	Post-2 vs. Post-3	39.31 ± 29.19 vs. 34.98 ± 18.16	0.358
	Post-3 vs. Post-4	34.98 ± 18.16 vs. 37.92 ± 19.01	0.489
	Pre-RT vs. Post-2	42.01 ± 25.79 vs. 39.31 ± 29.19	0.777
	Pre-RT vs. Post-3	42.01 ± 25.79 vs. 34.98 ± 18.16	0.329
	Pre-RT vs. Post-4	42.01 ± 25.79 vs. 37.92 ± 19.01	0.552
sST2	Pre-RT vs. Post-1	11.99 ± 3.89 vs. 12.43 ± 4.85	0.572
	Post-1 vs. Post-2	12.43 ± 4.85 vs. 11.91 ± 3.92	0.493
	Post-2 vs. Post-3	11.91 ± 3.92 vs. 10.57 ± 4.51	0.011
	Post-3 vs. Post-4	10.57 ± 4.51 vs. 10.72 ± 3.62	0.726
	Pre-RT vs. Post-2	11.99 ± 3.89 vs. 11.91 ± 3.92	0.914
	Pre-RT vs. Post-3	11.99 ± 3.89 vs. 10.57 ± 4.51	0.024
	Pre-RT vs. Post-4	11.99 ± 3.89 vs. 10.72 ± 3.62	0.052

**p*-values < 0.05 were considered statistically significant.

Table S2. Changes in metabolic activity on ^{18}F-FDG PET/CT before and after breast RT

			Mean ± SD	p-value*
30 Gy	Max uptake ratio	Pre-RT vs. Post-2	0.76 ± 0.14 vs. 0.91 ± 0.14	0.001
		Pre-RT vs. Post-4	0.76 ± 0.14 vs. 0.85 ± 0.13	0.035
		Post-2 vs. Post-4	0.91 ± 0.14 vs. 0.85 ± 0.13	0.111
	Mean uptake ratio	Pre-RT vs. Post-2	0.82 ± 0.11 vs. 0.95 ± 0.11	0.001
		Pre-RT vs. Post-4	0.82 ± 0.11 vs. 0.91 ± 0.14	0.007
		Post-2 vs. Post-4	0.95 ± 0.11 vs. 0.91 ± 0.14	0.226
47.5 Gy	Max uptake ratio	Pre-RT vs. Post-2	0.71 ± 0.15 vs. 0.85 ± 0.13	0.002
		Pre-RT vs. Post-4	0.71 ± 0.15 vs. 0.79 ± 0.14	0.007
		Post-2 vs. Post-4	0.85 ± 0.13 vs. 0.79 ± 0.14	0.135
	Mean uptake ratio	Pre-RT vs. Post-2	0.77 ± 0.17 vs. 0.94 ± 0.21	0.013
		Pre-RT vs. Post-4	0.77 ± 0.17 vs. 0.86 ± 0.27	0.151
		Post-2 vs. Post-4	0.94 ± 0.21 vs. 0.86 ± 0.27	0.186

*p-values < 0.05 were considered statistically significant.

Table S3. Changes in global longitudinal strain on echocardiography before and after breast RT

		Mean ± SD	p-value*
EF	Pre-RT vs. Post-2	66.31 ± 6.44 vs. 67.25 ± 6.05	0.643
	Post-2 vs. Post-3	67.25 ± 6.05 vs. 67.50 ± 6.19	0.903
	Post-3 vs. Post-4	67.50 ± 6.19 vs. 67.56 ± 5.92	0.972
	Pre-RT vs. Post-3	66.31 ± 6.44 vs. 67.50 ± 6.19	0.442
	Pre-RT vs. Post-4	66.31 ± 6.44 vs. 67.56 ± 5.92	0.498
GLS_Avg	Pre-RT vs. Post-2	-20.388 ± 2.47 vs. -19.538 ± 2.66	0.123
	Post-2 vs. Post-3	-19.538 ± 2.66 vs. -19.369 ± 2.96	0.768
	Post-3 vs. Post-4	-19.369 ± 2.96 vs. -19.269 ± 2.93	0.801
	Pre-RT vs. Post-3	-20.388 ± 2.47 vs. -19.369 ± 2.96	0.131
	Pre-RT vs. Post-4	-20.388 ± 2.47 vs. -19.269 ± 2.93	0.101
GLS_LAX	Pre-RT vs. Post-2	-20.20 ± 2.65 vs. -18.89 ± 2.61	0.015
	Post-2 vs. Post-3	-18.89 ± 2.61 vs. -19.33 ± 3.42	0.383
	Post-3 vs. Post-4	-19.33 ± 3.42 vs. -19.21 ± 3.38	0.823
	Pre-RT vs. Post-3	-20.20 ± 2.65 vs. -19.33 ± 3.42	0.261
	Pre-RT vs. Post-4	-20.20 ± 2.65 vs. -19.21 ± 3.38	0.156
GLS_A4C	Pre-RT vs. Post-2	-20.42 ± 2.44 vs. -19.43 ± 2.85	0.162
	Post-2 vs. Post-3	-19.43 ± 2.85 vs. -19.02 ± 2.87	0.573
	Post-3 vs. Post-4	-19.02 ± 2.87 vs. -19.47 ± 2.63	0.372
	Pre-RT vs. Post-3	-20.42 ± 2.44 vs. -19.02 ± 2.87	0.054
	Pre-RT vs. Post-4	-20.42 ± 2.44 vs. -19.47 ± 2.63	0.185
GLS_A2C	Pre-RT vs. Post-2	-20.54 ± 2.87 vs. -20.28 ± 3.04	0.689

Post-2 vs. Post-3	-20.28 ± 3.04 vs. -19.64 ± 3.49	0.411
Post-3 vs. Post-4	-19.64 ± 3.49 vs. -19.23 ± 3.34	0.555
Pre-RT vs. Post-3	-20.54 ± 2.87 vs. -19.64 ± 3.49	0.286
Pre-RT vs. Post-4	-20.54 ± 2.87 vs. -19.23 ± 3.34	0.154

**p*-values < 0.05 were considered statistically significant.
